# Practice at the County Infirmary

**Published:** 1882-03

**Authors:** A. W. Hagenbach

**Affiliations:** Assistant Superintendent Cook County Insane Asylum and Infirmary; Chicago


### ARTICLE V.

PRACTICE AT THE COUNTY INFIRMARY. By A. W. HAGENBACH, M.D., Assistant Superintendent Cook County Insane Asylum and Infirmary.

The Cook County Infirmary, with an average daily population of seven hundred inmates, with hospital accommodations for 110 patients (sixty males and fifty females) is located ten miles northwest of the center of the City of Chicago, and accommodates all the sick pauper patients not cared for in any of the other charitable institutions of Cook County. The accompanying tables will show the total number of cases treated in "Male Hospital Department" of Infirmary during the year 1881. In looking over the tables certain peculiarities present themselves which need some explanation. In the tables of "Medical Cases," two cases are entered as "anasarca, general;" as anasarca is not a disease *per se* but merely a symptom, it would seem necessary to state why they were entered as such in the tables. In both instances the patients, when admitted, presented no other prominent symptoms; neither of them were suffering with kidney or heart disease. The anasarca was regarded as a result of some previous disease, character unknown, which had left the blood alternated, so as to give rise to the dropsical effusion. The cases were accordingly entered as general anasarca, when admitted to the hospital, and so appear in the tables.

The second item calling for explanation is the large number of cases entered as "debility, general." By glancing over the tables of ages, it will be seen that 245 or over 50 per cent. of the total number of cases treated were over fifty years of age, while seventy-nine were over seventy years of age. A majority of the very old patients were admitted to hospital on account of general debility, they being too feeble to live in the other departments where they would have been compelled to leave their rooms to eat in a common dining-room. The ten cases of "Variola" developed in patients sent to hospital previous to the appearance of the eruption.

Another item needing an explanation is the large number of cases of angina pectoris, as compared with valvular lesions of the heart, there being six of the former and five of the latter. It must be remembered that the tables include all the cases treated during the year, each readmission being counted as a new case. Two patients with angina pectoris were each twice readmitted to hospital during the year, while patients with valvular lesions of heart, unless in an advanced stage, are rarely admitted to hospital at all. The large percentage of cases of "Phthisis Pulmonalis" is accounted for by the fact that but a limited number of these patients are admitted to the Cook County Hospital, the only charitable public hospital in Chicago. The frequency of diarrhœa among the inmates is due to previous abuse of the digestive organs, as well as the infirmary diet which, while containing all the proximate principles in sufficient quantities to keep up nutrition, does not present that variety which is conducive to perfect digestion and assimilation. The treatment generally followed in such cases is the administration of a cathartic, to remove all irritating matter from the intestines; castor oil generally answers better than any other cathartic, as it acts purely as an evacuant, and is non-irritating. The oil is generally followed by slightly astringent preparations and a more liberal diet. This simple treatment generally effects a speedy cure. In certain cases characterized by a want of intestinal tonicity, the following preparation has been used with marked success:

℞ Olei terebinth .................... ʒ ij.
Tinct. opii .................... ʒ iii.
Syr. krameriæ .................... ℥ ij.
Aquæ pura ad .................... ℥ iv.
M. Emulsify.
Sig.—Teaspoonful every 3 or 4 hours.

I have also found this mixture, very useful in certain cases of diarrhœa, among the chronic insane. The promiscuous use of astringents is useless, and capable of doing harm by irritating the mucous lining of stomach and intestines.

The only items in tables of "Surgical Cases" calling for special notice are the large number of cases of fractures, and the case of "Strangulated Inguinal Hernia." The fractures of femur, of neck of femur, ribs, and of tibia and fibula, were admitted immediately after the accidents by which they were caused, and were treated here throughout; the remaining cases were transferred from Cook County Hospital with dressings applied. The patient with strangulated inguinal hernia was admitted to hospital department for the second time. The hernia could not be reduced either time, even after the patient was completely anæsthetized. As there were no alarming symptoms present, he was placed in bed, and ice bags applied continuously over tumor for twelve hours, when it was reduced without difficulty. The tables of "The Cause of Death" call for no special explanations.

*Nativity of Patients treated in Cook County Infirmary—Male Department—during the year* 1881.‡

| | | | |
|---|---|---|---|
| United States, white | 75 | Germany | 111 |
| " " black | 14 | Holland | 1 |
| | —— 89 | Ireland | 148 |
| Batavia | 1 | Norway | 20 |
| Belgium | 1 | Russia | 2 |
| Bohemia | 13 | Scotland | 7 |
| Canada | 10 | Sweden | 22 |
| Denmark | 7 | Switzerland | 2 |
| England | 25 | | —— |
| France | 3 | Total | 462 |

*Ages of Patients treated in Cook County Infirmary—Male Department—dur- the year* 1881.

| | | | |
|---|---|---|---|
| From 12 years to 20 years | 4 | From 60 years to 65 years | 32 |
| " 20 " 25 " | 19 | " 65 " 70 " | 43 |
| " 25 " 30 " | 26 | " 70 " 75 " | 27 |
| " 30 " 35 " | 39 | " 75 " 80 " | 26 |
| " 35 " 40 " | 40 | " 80 " 85 " | 20 |
| " 40 " 45 " | 50 | " 85 " 90 " | 5 |
| " 45 " 50 " | 39 | " 90 " 95 " | 1 |
| " 50 " 55 " | 39 | | —— |
| " 55 " 60 " | 52 | Total | 462 |

*Statement of Medical Cases treated in Cook County Infirmary—Male Department—during the year* 1881.

| | | | | | |
|---|---|---|---|---|---|
| *General.* | | | *Nervous System.* | | |
| Ague, tertian | 6 | | Apoplexy, cerebral | 1 | |
| Alcoholism | 2 | | Ataxia, locomotor | 3 | |
| ‡ Anasarca, general | 2 | | Catalepsy | 1 | |
| Anemia | 5 | | Dementia | 6 | |
| ‡ Debility, general | 55 | | Epilepsy | 3 | |
| Fever, remittent | 1 | | Hemiplegia, right | 13 | |
| " scarlet | 1 | | " left | 12 | |
| " typhoid | 1 | | Mania, acute | 2 | |
| Lumbago | 1 | | Myelitis | 1 | |
| Opium habit | 1 | | Paralysis | 2 | |
| Pleurodynia | 2 | | " agitans | 3 | |
| Pyemia | 1 | | " arm | 2 | |
| Rheumatism, chronic | 8 | | " both extremities | 1 | |
| " articular acute | 4 | | Paraplegia | 3 | |
| " syphilitic | 6 | | | —— | 53 |
| ‡ Variola | 10 | | *Heart.* | | |
| | 106 | | ‡ Heart, valvular lesions of | 5 | |
| * Malingerer | 3 | | Angina pectoris | 6 | |
| | —— | 109 | | —— | 11 |
| *Lungs.* | | | *Intestinal Tract, &c.* | | |
| Asthma | 11 | | Cirrhosis, liver | 4 | |
| Bronchitis | 32 | | Coeliagra | 1 | |
| Emphysema | 1 | | ‡ Diarrhœa | 23 | |
| ‡ Phthisis pulmonalis | 49 | | Dysentery | 1 | |
| Pleuritis | 4 | | Gastro-enteritis | 4 | |
| Pleuro-pneumonitis | 2 | | Tabes Mesenterica | 1 | |
| Pneumonitis | 7 | | | —— | 34 |
| | —— | 106 | *Kidneys.* | | |
| | | | Bright's disease | | 3 |
| | | | Total | | 316 |

*Statement of Surgical Cases treated in Cook County Infirmary—Male Department—during the year* 1881.

| | | |
|---|---|---|
| *Abscess.* | | |
| Abscess, leg from erysipelas | 3 | |
| " " " injury | 2 | |
| " Axilla | 1 | |
| * " Mastoid cells | 1 | |
| Carbuncle over Mastoid process | 1 | |
| | | 9 |
| *Affections of Bones and Appendages.* | | |
| Caries, elbow joint | 1 | |
| Exostosis, tarsal bones | 1 | |
| ‡Fracture, femur | 1 | |
| " " neck of | 1 | |
| " humerus | 2 | |
| " patella | 2 | |
| " "Potts" | 2 | |
| " ribs | 2 | |
| " scapula | 1 | |
| " tibia and fibula | 2 | |
| " ulna | 1 | |
| Hip joint, disease | 1 | |
| Knee, dislocation | 1 | |
| Necrosis, femur | 1 | |
| *Osteo sarcoma, femur | 1 | |
| Paronychia | 1 | |
| Spinal curvature | 2 | |
| Sprain, ankle | 1 | |
| Tarsus, crushed | 2 | |
| | | 26 |
| *Affections of Integument.* | | |
| *Combustio, dermatitis | 1 | |
| Erysipelas, traumatic | 9 | |
| Frost bite | 5 | |
| Scrotum, gangrene of | 1 | |
| Stumps, ulcerated | 4 | |
| *Ulcers, indolent | 17 | |
| " inflamed | 1 | |
| " syphilitic | 1 | |
| " varicose | 11 | |
| | | 50 |
| *Specific.* | | |
| Cancer, rectum, scirrhus | 1 | |
| " neck, encephaloid | 2 | |
| " pylorus, scirrhus | 2 | |
| Syphilis, primary | 9 | |
| " secondary | 7 | |
| * " tertiary | 2 | |
| Gonorrhœa | 4 | |
| | | 27 |
| *Not Classified.* | | |
| Cataract, senile | 4 | |
| Conjunctivitis | 4 | |
| Contusions | 9 | |
| Hernia, double inguinal | 2 | |
| ‡ " inguinal strangulated | 1 | |
| Hydrocele | 2 | |
| Prolapsus, rectum | 2 | |
| Stricture, urethra | 2 | |
| Synovitis | 6 | |
| Trichiasis | 2 | |
| | | 34 |
| Total | | 146 |

*Nativity of Patients who died in Cook County Infirmary—Male Department—during the year* 1881.

| | | |
|---|---|---|
| United States, white | 18 | |
| " " black | 2 | |
| | | 20 |
| Belgium | | 1 |
| Bohemia | | 6 |
| Canada | | 4 |
| Denmark | | 1 |
| England | | 3 |
| France | | 2 |
| Germany | | 30 |
| Ireland | | 31 |
| Norway | | 2 |
| Sweden | | 7 |
| Switzerland | | 2 |
| Scotland | | 2 |
| Total | | 111 |

*Ages of Patients who died in Cook County Infirmary—Male Department—during the year* 1881. ‡

| | |
|---|---|
| From 15 years to 20 years | 1 |
| " 20 " 25 " | 3 |
| " 25 " 30 " | 4 |
| " 30 " 35 " | 12 |
| " 35 " 40 " | 6 |
| " 40 " 45 " | 7 |
| " 45 " 50 " | 10 |
| " 50 " 55 " | 9 |
| From 55 years to 60 years | 11 |
| " 60 " 65 " | 9 |
| " 65 " 70 " | 16 |
| " 70 " 75 " | 9 |
| " 75 " 80 " | 7 |
| " 80 " 85 " | 5 |
| " 85 " 90 " | 1 |
| " 90 " | 1 |
| Total | 111 |

*Cause of death of Patients in Cook County Infirmary—Male Department—during the year* 1881.

| *Immediate Cause.* | *Complications.* | | |
|---|---|---|---|
| Alcoholism | | 1 | |
| " | Dysentery | 1 | |
| | | — | 2 |
| Apoplexy, cerebral | Hemiplegia, right side | 1 | |
| " " | Old age | 1 | |
| " " | Hemiplegia | 1 | |
| | | — | 3 |
| Asthma | Chronic bronchitis | | 5 |
| Bronchitis | | 3 | |
| " | Acute tonsilitis | 1 | |
| " | Old age | 2 | |
| | | — | 6 |
| Bright's disease | Valvular lesions of heart | | 1 |
| Cancer, pylorus, scirrhus | | 2 | |
| " neck, encephaloid | | 1 | |
| | | — | 3 |
| Cirrhosis, liver | Icterus, peritoneal dropsy | 1 | |
| " " | Peritoneal dropsy | 3 | |
| | | — | 4 |
| Combustio, dermatitis | | | 12 |
| Debility, general | Dementia | 2 | |
| " " | Hemiplegia | 3 | |
| " " | " right side | 1 | |
| " " | " left " | 1 | |
| " " | Old age | 9 | |
| " " | Suppurating buboes | 1 | |
| " " | Ulcers, both legs | 1 | |
| " " | Paralysis, both extremities | 1 | |
| | | — | 19 |
| Dementia | Hemiplegia, right side | 1 | |
| " | Old age | 1 | |
| " | Hemiplegia | 1 | |
| | | — | 3 |
| Diarrhœa | | 5 | |
| " | Cystitis, chronic | 1 | |
| " | Dementia | 2 | |
| " | Hemiplegia | 1 | |
| " | Melancholia | 1 | |
| " | Old age | 4 | |
| " | Paralysis both extremities, partial | 1 | |
| " | Ulcers, legs | 1 | |
| " | Hemiplegia, right side | 1 | |
| | | — | 17 |
| Fever, scarlet, malignant | | | 1 |
| " typhoid | | | 1 |
| Gastro-enteritis | | | 1 |
| Hemorrhage, pulmonary | Phthisis pulmonalis | | 1 |
| Heart, aortic and mitral lesions | Enlargement by dilatation | | 1 |
| Inanition | Bronchitis | | 1 |
| Mania, acute | Ulcerated stump | | 1 |
| Myelitis | Paraplegia and bed sores | | 1 |
| Osteo Sarcoma | | | 1 |
| Paralysis Agitans | | | 1 |
| Phthisis pulmonalis | | 15 | |
| " " | Anemia | 1 | |
| " " | Bronchitis | 1 | |
| " " | General anasarca | 1 | |
| " " | Diarrhœa | 2 | |
| " " | Angina pectoris | 1 | |
| " " | Hypertrophy of heart | 1 | |
| " " | Pleuritis circumscribed | 1 | |
| " " | Syphilis, secondary | 1 | |
| | | — | 24 |
| Pleuritis circumscribed | Phthisis pulmonalis | | 1 |
| Pleuro pneumonitis, left side | | | 1 |
| Pneumonitis, left lower lobe | | 1 | |
| " | Hydro-thorax | 1 | |
| " | Phthisis pulmonalis | 2 | |
| " | Bronchitis | 1 | |
| | | — | 5 |
| Pyemia from erysipelas | | 1 | |
| " " abscess of mastoid cells | | 1 | |
| | | — | 2 |
| Rheumatism, chronic | Bronchitis | | 1 |
| Suicide by hanging | | | 1 |
| Syphilis, secondary | | | 1 |
| Tabes Mesenterica | | | 1 |
| | Total | | 111 |

*Recapitulation of Cause of Death.*

| *Immediate Cause.* | | *Complications.* | |
|---|---|---|---|
| Alcoholism | 2 | Anemia | 1 |
| Apoplexy | 3 | Bronchitis | 9 |
| Asthma | 5 | Cystitis | 1 |
| Bronchitis | 6 | Bed sores | 1 |
| Bright's disease | 1 | Diarrhœa | 2 |
| Cancer | 3 | Dropsy | 4 |
| Cirrhosis | 4 | Dysentery | 1 |
| Combustio dermatitis | 1 | Dementia | 4 |
| Debility | 19 | Heart lesions | 4 |
| Dementia | 3 | Icterus | 1 |
| Diarrhœa | 17 | Melancholia | 1 |
| Fever, scarlet | 1 | Old age | 17 |
| Fever, typhoid | 1 | Paralysis | 13 |
| Gastro enteritis | 1 | Phthisis pulmonalis | 4 |
| Hemorrhage, pulmonary, immediate | 1 | Tonsilitis | 1 |
| Heart, lesions of | 1 | Syphilis | 2 |
| Inanition | 1 | Ulcers | 3 |
| Mania, acute | 1 | Hydro-thorax | 1 |
| Myelitis | 1 | Pleuritis | 1 |
| Osteo sarcoma | 1 | Not specified | 40 |
| Paralysis agitans | 1 | | |
| Phthisis pulmonalis | 24 | Total | 111 |
| Pleuritis | 1 | | |
| Pleuro pneumonitis | 1 | | |
| Pneumonitis | 5 | | |
| Pyemia | 2 | | |
| Rheumatism | 1 | | |
| Suicide | 1 | | |
| Syphilis | 1 | | |
| Tabes Mesenterica | 1 | | |
| Total | 111 | | |

* Cases reported in this article.

† I would acknowledge the valuable assistance rendered by Mr. T. Sydney, in preparing the tables.

‡ See explanations of tables.

*Malingerers.*—Malingerers are frequently met with in the infirmaries of large cities, among a class of shiftless men who, after spending all their earnings during the summer, will resort to any disreputable means to gain admission to some charitable institution during the winter months. Physicians who have had experience in dealing with paupers generally recognize such customers at a glance, but even they are sometimes deceived.

*Case.*—Only a few months ago, an apparently healthy man presented himself at the dispensary department, asking for an excuse from work on account of paralysis, which he claimed affected his right arm. His general appearance, and the position of the member, which was held closely to the side, led me to suspect him of being an impostor. Upon trying to elevate the arm, I found the muscular resistance so great that I experienced some difficulty in raising it to a horizontal position. When released, it descended with considerable force to its original position. Asking an attendant to hold the arm in a raised position, I directed the patient's attention to an eruption on his face, at the same time giving the nurse a nod to release the arm, which remained stationary for several moments after the support was gone, when he suddenly jerked the arm downward with great force, to the amusement of several inmates who were present during the examination. It is perhaps needless to state that after being detected he at once went to work, and never afterward attempted to "sham Abraham." The ignorance of symptomatology displayed by this patient is characteristic of the usual pauper malingerer, and the chief element that leads to immediate detection. An exaggeration of symptoms is almost daily encountered among the older inmates, who know that sickness will assure an excuse from work, as well as special diet. While malingerers generally are readily detected, some will mislead the most experienced. A remarkable case came under my observation in 1877, in the female hospital department of the infirmary.

*Case.*—Maria B., æt. about fifty, complaining of severe pain in head and back, also of loss of power in extremities, remained in bed for several years, the dread of the nurses, and the pest of the attending physicians. She became so exacting in her demands that Dr. J. Lawless, the assistant superintendent, had her removed to an adjoining building where she could be more isolated, and would necessarily receive less attention than in the hospital department. Judge of our surprise when, on the following morning, her bed was empty, and Maria was nowhere to be found, as she had left during the night. It might be claimed that a proper diagnosis should have been made of the case long before; but to show that the physicians at the infirmary were not the only ones deceived by her, it might be well to state that several months later, while passing through the wards of the Cook County Hospital, Dr. Lawless found her snugly in bed, as troublesome as ever. On informing the physicians of his experience with her at the infirmary, she was at once dismissed. A few days later she was seen on the railroad, walking toward Milwaukee, where I have no doubt she succeeded with her impositions, as nothing has been heard of her since. Of the three cases recorded in the tables, but one needs special notice.

*Case.*—T. T.,* æt. forty-two, admitted October 20, 1881.

Ever since discharged from hospital department, where he had received special attention, he tried to gain a readmission, claiming that since dismissed he was subject to fits. His continued effort to gain readmission, as well as the contradictory character of his story as told at different times, led us to suspect that the fits were feigned. He was sent to hospital, the nurse receiving instructions carefully to notice his general deportment. I was called to see the case by Dr. A. Baldwin, house physician, a few days after his admission, while suffering (?) from a very severe attack. I found him lying in bed, foaming at the mouth, kicking and twisting himself into innumerable shapes. The muscular contractions were limited almost entirely to the right side. Owing to the violent exertions, he was bathed in perspiration. Pulse 80. Pupils normal in size, and responding readily to light. To confirm our suspicions concerning the nature of the fits, the administration of chloroform was discussed in the presence of the patient. A small quantity of chloroform poured on a towel was held over his mouth and nose. The fits immediately grew milder, and ceased suddenly after three or four inspir-

* This is the same patient who had previously been treated in hospital, suffering with perineal abscess and gangrene of scrotum.

ations, and have not since returned, the patient working steadily in the hospital kitchen. The treatment followed in such cases is very simple and effective. The malingerer is at once dismissed or set to work.

*Osteo Sarcoma.*—Case. J. F., æt. seventeen, admitted May 26, 1881. Previous history unknown, as the patient was unable to give any account of himself, or even the address of his friends. He was emaciated, and acted as if in great pain, crying out whenever touched. He would frequently moan for hours; then suddenly cry out, as if in great agony. He spoke of the pain as "shooting through the leg." His entire left thigh was enormously enlarged from knee to hip joint, measuring at the thickest part 31⅝ inches in circumference. Circumference of body on a line with umbilicus 27½ inches. The integument of thigh was of a light stone color, and greatly stretched, so that the sweat pores were widely separated. The superficial fascia was ruptured in several places similar to what is seen beneath the abdominal integument toward the close of pregnancy. The thigh was hard to the touch, excepting a few small spots which were quite soft, giving to the finger the sensation of some deep seated semi-fluid substance. The superficial veins of thigh were enormously enlarged and tortuous, as were also the superficial epigastric, and superficial circumflex iliac veins on lower portion of abdomen on the side corresponding with the tumor. No ulcerations had taken place, the integument being intact throughout. The surface of the tumor presented numerous elevations and depressions, giving it a somewhat lobulated appearance. The leg was greatly atrophied, the contrast between it and the thigh was so marked, that it was almost impossible to conceive of the former as ever having supported the latter. So great was the weight of the enlarged thigh that he was utterly unable to raise it from the bed, and only with great difficulty was he able to change its position slightly. His bowels were loose throughout. He failed rapidly and died of exhaustion June 3, 1881. The only treatment adopted was the administration of morphine to relieve pain, large doses affording but slight relief. Good diet and cleanliness were prescribed throughout. At a post-mortem held twenty hours after death the general appearance of the thigh was the same as already described. An incision was carried along the anterior median line of the thigh dividing the integument and superficial fascia, exposing the muscles spread out very thin, and so pale that I at first mistook them for the deeper fascia. Immediately beneath the muscular layer a dense white membrane was found, enveloping the entire growth. This membrane was nearly a line in thickness and very dense, giving a sensation to the hand in dividing similar to that experienced in cutting cartilage. Beneath this membrane the entire tumor was found distended with a granular lardaceous matter, and a small quantity of liquid resembling glycerine in color and consistency. The tumor was divided into innumerable cells or spaces by a thin shining membrane, in certain portions of which small deposits of osseous matter were found. The spaces were of all sizes, containing from several drachms to a quart or more of the lardaceous matter. When the bone was exposed it was discovered that the entire thickness of the shaft of the femur was honey-combed, the compact tissue in some places had been completely destroyed by an ulcerative process, the cells of the cancellous tissues were enlarged.*

*Combustio*, *Dermatitis*.—Case. H. C., cook; while pouring kerosene out of a can containing nearly a gallon of the oil on some ignited wood in a cook-stove, the kerosene in the can caught fire, caused an explosion to take place, throwing the burning contents all over him, burning him severely about the face, upper and lower extremities, and a portion of the trunk, involving fully one half of entire surface of the integument in the burn. Several pieces of integument were unavoidably detached in removing the clothing to which it had become adherent in places. Severe nervous shock was the chief symptom following the accident. The extremities were cold. Pulse small and compressible. Temperature 97½ F. Lime-water and linseed-oil dressings were applied continuously and stimulants freely administered. A few hours after the accident he commenced to complain of pain in epigastric region, followed by vomiting. The stomach became very irritable, at first rejecting liquid food administered, and several hours later would not retain a single drachm of water.

*For a very interesting case tending to show hereditary nature of osteo sarcoma, see "Gibbon's Surgery," Vol. I.

He died 92 hours after the accident. A post-mortem was held 16 hours after death and the following pathological conditions were noted. Severe congestion of all the internal organs examined, especially the stomach and intestines. Several small ulcers were found in duodenum, two were quite deep, involving the muscular coat. I have no doubt had the patient lived several days longer, they might have caused complete perforation. The case illustrates the acute character of the duodenal ulcerations, which 92 hours after the accident had already destroyed the mucous and involved the muscular coat of the intestines. From the appearance of the deeper ulcers we were led to believe them of at least 48 hours duration previous to death. If correct in this estimation the ulcerative process was established inside of two days after the accident. I have been unable to find any satisfactory explanation of the cause of the ulcerations, notwithstanding the theory of the arrest of the exhalent functions of the injured integuments, etc.

*Abscess of Mastoid Cells and Pyœmia.*—Case. H. S., aged 40, was admitted to hospital department February 12, 1881, on account of "general ill health." Several weeks after admission he commenced to complain of pain in left ear, followed in a few days by a free discharge of pus. Thorough cleansing with warm water and the use of a slightly astringent solution was followed by marked improvement, the pain and discharge disappearing almost entirely. Several weeks later the pain returned, also severe pain in mastoid region was complained of. Hot fomentations were applied back of the ear for several days without affording relief from pain. Several ounces of blood were removed by making free incisions over mastoid process, affording partial relief, but as the pain soon returned with increased severity, and suspecting the presence of pus in the mastoid cells I concluded to afford it a free outlet. Several holes were drilled into the mastoid cells with an ordinary bone-drill and a button of bone removed, afterward breaking down the bony septa between the cells. The entire cavity was found filled with ichorous pus, which was removed, and the cavity washed out with a five per cent. solution of carbolic acid. The operation afforded almost instant relief from pain. The general health of the patient, however, became more and more impaired, and he experienced several severe chills the day following the operation. Mucous râles were also found present in both lungs. Large pus cavities formed in both hands, right axilla, right forearm, and later upon back; when opened they discharged large quantities of poorly conditioned, offensive pus. His appetite completely disappeared. Temperature ranged from 101° to 104° F. He lingered for ten days in a miserable condition and died of "exhaustion accompanying pyæmia" April 4, 1881. Ferruginous and vegetable tonics, good diet and stimulants were freely used.

*Tertiary Syphilis.*—The rules governing the admission of patients to the charitable institutions supported by this county prohibit the admission of patients suffering with primary syphilis. The occasional exceptions made to the rules will account for the nine cases recorded in the tables. The City of Chicago has made no provisions whatever for this unfortunate class of patients, and if all were admitted to the infirmary, the hospital department would accommodate no other patients. As the unfortunate pauper suffering from syphilis has no means of procuring treatment during the primary stage of the disease, cases are not unfrequently admitted with secondary, or even tertiary symptoms who have received no previous treatment. One of the worst cases of tertiary syphilis ever admitted was discharged during the present year, relieved.

*Case.*—W. P., sailor, aged 45, was admitted January 12, 1879. He had contracted syphilis about two and a half years previous to his admission. For reasons already stated he was unable to gain admission to any of the charitable institutions supported by this county, until he presented such a loathsome appearance, that to rid the city of his presence, he was finally sent to the infirmary. When admitted, he was covered with ulcers, varying in size from that of a split-pea, to several inches in diameter. Numerous patches of pustules were also found, beneath which destructive ulceration was going on. One very deep ulcer was located immediately over the sternum, exposing fully an inch of the gladiolus to view. Both feet (especially the right), were masses of dried pus, scabs and ulcers, bearing but slight resemblance to a human member. One of the attendants, when he saw the patient for the first time, was so struck by his appearance, that he named him "skinny," an appellation that hung to him until discharged. The great destruction of integument should have suggested skinless as a more expressive term. The treatment, at first, consisted in the application of hot fomentations wherever the pustules had not broken down so as to expose the ulcers beneath. Hot fomentations were also used to soften all hardened accumulations. When all the ulcers were exposed to view, it was found that they involved fully a third of the entire surface of integument. A dressing of simple cerate was applied to ulcers and the following mixture prescribed:

| | | |
|---|---|---|
| ℞ | Hydrarg chl. coros.. .. | gr. iss. |
| | Potas. iod...... ...... | ℥ ij. |
| | Syr. sars. comp......... | ℥ iii. |
| | Aquæ Pura ad...... ... | ℥ viii. |

Sig. Tablespoonful three times a day.

This treatment was followed for several months without any apparent improvement. The general health of the patient commencing to fail, iron, and later, cod-liver oil were prescribed. Various other preparations of mercury were at times substituted for the hydarg. chlor. corrosivum, in the above mixture. The improvement was very slow, when in August, 1880, I determined to use medicated steam baths in connection with internal medication. Iodine, sulphur, and carbolic-acid baths were alternately administered. After a few baths a marked improvement was noticed in the appearance of the ulcers; which were completely healed at the end of four months. His protracted confinement in bed had caused muscular rigidity of flexor muscles of both thighs to take place, so that the leg could not be fully extended. With returning health and exercise this complication gradually disappeared, he being able to walk with the aid of an ordinary walking-stick when discharged from the hospital, July 30, 1881. The surface of the integument not involved by the ulcers presented a pale, harsh appearance, and a large quantity of epithelial scales were removed every day. The general health of the patient at times was greatly impaired, when specific remedies were discontinued for short periods. With the exception of these intervals, perhaps three months in all, he was under "mixed," iodine and mercury, treatment until discharged. This case is reported to illustrate what persistent treatment will accomplish even in the most aggravated cases of tertiary syphilis, with especial reference to the efficacy of medicated steam baths, which I have found very useful in a number of cases where internal remedies had proven of but little value.

*Carbolic Acid Poisoning.*—Case. *Mrs. D., German, aged 35, for several years an inmate of the Cook County Hospital, for insane suffering from "chronic mania;" on October 19, 1881, succeeded in gaining access to a bottle of full strength carbolic acid, of which she swallowed a quantity, estimated at from one to two ounces. The female attendant on the ward, who saw her almost immediately afterward, said the patient "appeared as usual" and informed her that she had taken a dose of "medicine." While leading the patient to her room she suddenly became "drawn together," her hands clenched and strongly flexed upon forearms. Medical aid was at once summoned. Dr. H. Gray, who attended the call within ten minutes of the accident, found her unconscious, lids partially closed, lips and tongue white, face of a dark hue, and marked venous congestion. The odor of carbolic acid was very apparent the moment he approached the patient. About a pint of olive oil was injected into stomach, removed in a few minutes and a second pint of oil injected, the patient expiring a few moments later. The breathing at first was sighing, later stertorous. Temperature and pulse not noted. At a post-mortem held twenty hours after death, the odor of carbolic acid was still quite noticable about the mouth of the corpse. Lower half of entire body of a uniform red, a lighter hue than usually observed in post-mortem statis. The tongue and roof of mouth showed evidence of the action of some corrosive substance. The mouth and tongue were white, excepting where the mucous membrane had been detached so as to expose the deeper tissues, which were red and congested. No other abnormal appearances were detected by external examination. The contents of the thorax and abdomen were next examined. The stomach was found distended with air and the oil injected previous to death, and of a deep red color in patches on its external surface. The entire intestines were

*This patient was not an inmate of the Infirmary proper, but of Insane Hospital adjoining and under the same medical management.

congested, the smallest vein, when cut into, was followed by the escape of liquid blood of a brighter red than ordinary venous blood. The stomach, and ten inches of the duodenum were removed and laid open for further examination, when the mucous membrane on posterior surface of stomach, near the great cul de sac, was found hardened and of a dark brown color. This I regarded as the spot where the concentrated acid first came in contact with the mucous lining of the stomach, the hardening being due to the coagulation of the albumen of the membrane. Over the remainder of the stomach the mucous membrane was almost completely destroyed, so as to expose the muscular coat smooth and shining beneath. This destruction of the mucous membrane I do not regard as due to post-mortem digestion,* as the gastric juice must have been changed by the acid, and there was no possible mistaking the direct action of some corrosive substance. So powerful was the action of the acid that several arterioles had ruptured, and the expelled blood upon coming in contact with the acid had coagulated. The portion of the duodenum removed was highly congested, and of a uniform red color. The œsophagus, when spread out, presented a striped appearance, alternate red and pale lines extending its entire length. This appearance was regarded as due to the arrangement of the mucous membrane in longitudinal folds. Two blue spots about the size of a small bean were noticed beneath the mucous membrane, immediately above the œsophageal opening in diaphragm ; when cut into they were found to contain dark coagula. As these spots contained the only coagulated blood found in entire body, I regarded them as due to some injury to the œsophagus, previous to death, possibly the arrest for a time of some foreign body accidentally swallowed.† The liver and lungs were distended with liquid blood. The heart was completely empty, and a small deposit of tubercle was detected in the apex of left lung.

*Perineal Abscess and Gangrene of Scrotum.*—Illustrating the rapidity with which extensive destruction of scrotal tissues are repaired.

---

*The digestion of the walls of the stomach that sometimes takes place after sudden deaths in vigorous subjects.

†Foreign bodies are quite frequently arrested in œsophagi of the insane. See my article on "Surgery among the Insane," also "Foreign Bodies in Surgery."—*Poulet.*

*Case.*—* T. T., aged 42, suffering for may years with facial neuralgia, presented himself at the out-department of the Cook County Poor House, Oct. 4, 1880, complaining of severe pain in scrotum and testes. Upon examination, found perineum hard and swollen, with erysipelas involving the entire scrotum. He was transferred to the hospital department, and hot water dressings were ordered to be applied continuously.

Oct. 5th. Scrotum swollen to twice its size, and erysipelas extending over lower part of abdomen. The penis is greatly distended, with effusion under integument. Hot water dressings continued, and the following preparation prescribed:

℞ Quiniæ Sulphatis.........ʒj

Tinct. Ferri Chloridi.....ʒij

Syr Tolutani..................℥j

Aquæ Puræ aa. ........... M

Sig.—Teaspoonful 3 times a day.

Opium in sufficient quantities to relieve pain.

Oct. 6th. The erysipelas extending higher over abdomen. Scrotum enlarged to the size of the fœtal head. The swelling of perineum enlarging and very painful. No fluctuation can be detected.

Oct. 7th. The case was seen to-day, by Drs. Wilde, Cohen, Bessler and Thiely, of Chicago, who advised a continuation of the treatment adopted, and expressed an unfavorable prognosis, as all the symptoms present pointed to extensive destruction of scrotal tissue and death from exhaustion, or septicæmia. Opening the perineal abscess, as soon as the pus approached the surface, was recommended.

Oct. 8th. About 5 o'clock this A.M. the abscess opened spontaneously near the center of the perineum, discharging a large quantity of poorly-conditioned, offensive pus.

Oct. 10th. Circulation in anterior surface of scrotum entirely arrested. Urine escaping through opening in perineum, an elastic catheter was passed into the bladder without difficulty.

Oct. 14th. Line of demarkation commencing to form, the mortification involving greater part of anterior surface and base of scrotum. Carbolized linseed poultices were now applied instead

* This case was reported by me in an article on "Surgery among the Insane." Read before the West Chicago Medical Society, and published in the *Journal of Nervous and Mental Disease*, Vol. viii. No. 1. January, 1881.

of hot-water dressings; the slough at once commenced to separate, and was completely removed by the 19th, when nearly half of the scrotal tissue was gone, both testicles being plainly exposed to view, the left protruding partially through the opening. After pressing the testicle upward, applied strips of adhesive plaster, bringing together the opposite sides of the wound, and affording support to the testicles.

Oct. 24th. Adhesive straps have been applied daily since the 19th; opening about one-half former size. Opening in perineum almost closed. Patient can retain urine for several hours, and but little escapes through opening in perineum.

Oct. 30th. Healing very rapidly, the opposite side of the wound remaining in contact without strapping; testicles in normal position.

Nov. 15th. Wound healed, and patient discharged cured in less than a month after the destruction of nearly half of the scrotal tissue.

*Ulcers.*—Of all the surgical cases admitted to this institution, these are by far the most numerous. The city hospitals being crowded with patients, suffering from more acute affections, rarely admit more of these cases than are required for clinical purposes; hence, the large percentage treated at the infirmary. Thirty cases of ulcers of inferior extremities are recorded in the tables, but this number does not include one-half of the cases actually treated, as only those it is impossible to care for in other apartments are ever transferred to the hospital. Some of the ulcers are very large, extending in some instances from the ankle almost to the knee, and involving nearly entire circumference of leg. The treatment usually pursued is: 1st. To place the patient in recumbent position; 2d. The application of hot fomentations until parts are thoroughly cleansed; 3d. A thorough application of caustic, to stimulate parts to healthy action; 4th. The use of strips of adhesive plaster to support edges of ulcer. Ointment or salves are very rarely used. Skin grafting is resorted to in suitable cases, after the granulations present a healthy appearance. I have found the greatest success in grafting, by applying the bits of integument immediately upon the granulations, without much, if any, scarification of the surface of the ulcer. I succeeded a few weeks ago in growing six out of nine pieces, grafted in this manner on a patient who had undergone several previous unsuccessful operations, two of which were performed by a Chicago surgeon of note. The patient had but little faith in the way I performed the operation, and informed me that it was "very difficult to make them take" on him, as Dr. —— had tried it upon two occasions, each time scarifying the granulations until the blood trickled down the leg, and still they refused to grow. The deep incisions causing a free escape of blood, glazing entire surface of ulcer, I regard as the chief, if not sole reason of the failure of previous operations. Attention must also be paid to the general health of patients with every variety of ulcers.

*Varicose Ulcers.*—So far as the ulcers are concerned, the same general treatment is followed as in the indolent variety, with the additional application of a roller, or rubber bandage, to compress the enlarged veins. It was formerly my custom to ligate the veins in a large percentage of the cases that came under my care, affording permanent relief in several cases. I have had but one case in which the operation was accompanied, or followed by any alarming symptoms.

*Case.*—J. R., aged 42, was transferred from the Cook County Hospital, where he had undergone two previous operations; six pins had been inserted each time. When admitted he desired to have the treatment continued, claiming to have derived great relief from the previous operations. As quite a number of varicose veins still remained on both legs, I inserted five pins under the most prominent of the veins, crossed them with twisted silk suture, and allowed them to remain until they had ulcerated completely through the inclosed tissues. He was then allowed several weeks to recuperate, when five pins more were inserted and crossed with twisted suture as before. Two of the pins had already ulcerated completely through, when the patient commenced to complain of pain in the head and jaws, followed by thickness of speech and difficulty in swallowing, especially fluids. The two remaining pins were removed, and linseed poultices applied over both legs. He suffered intense pain, and rapidly grew worse, the jaw became firmly locked, and later the muscles of neck and back became involved in the spasm. The slightest cutaneous irritation was followed by severe tetanic contractions. Growing rapidly worse, he died forty-eight hours after the appearance of alarming symptoms. The treatment consisted in the administration of chloral hydrate and opium, with chloroform inhalations, for several hours previous to death, to control the violent spasms. An enema of tobacco was also administered, and followed by a free discharge from the bowels. I was inclined to ascribe the exciting cause of the tetanus to nervous irritation, caused by including a nerve of considerable size in some one of the last five ligatures.

---

COMMUNICATION OF SYPHILIS BY SKIN-GRAFTING. — Dr. Deubel communicated the following case to the Société Médical des Hopitaux: A man, aged forty-nine, who had never contracted venereal disease, became, in January, 1881, the subject of gangrenous erysipelas of the thigh, which was attended with a large ulceration, having its starting point in the superficial ulceration of some hemorrhoids, that, except at some isolated points, refused to cicatrize. On March 7, forty-five dermo-epidermic grafts, furnished by five persons, were inserted, and thirty-three of these contracted adhesions; and twenty other grafts taken from seven persons, aged from twenty to forty years, were placed on another part of the wound on the 23d, thirty of the number retaining their vitality. Grafts from the mucous membrane of the mouth of a rabbit were also employed, and on March 23 forty new grafts, taken from persons aged from twelve to fifty-four years, were applied. Cicatrization went on satisfactorily until April 5, when ulceration commenced in the now almost cicatrized wound where the first grafts had been planted, and soon destroyed the cicatrization. The grafts applied on the second occasion did not ulcerate, but became pale and fell off. The new ulceration had a syphilitic aspect; but the man's wife, who had nursed him, having also been attacked with erysipelas which proved fatal, and a lodger suffering from lymphangitis, it was concluded that the whole had arisen from infectious causes due to the very unsanitary condition of the house in which they all resided. The ulcerations improved on being touched with nitrate of silver, but new ones kept appearing during the next three months, and ten weeks after the first application of the grafts the skin and scalp became the seat of syphilitic eruption, and some weeks later the mucous membrane of the mouth was affected. A mercurial and iodide treatment was put into force, and eight months after the first appearance of the erysipelas the breach of surface became entirely cicatrized. It turned out that a son, who had furnished some of the grafts, was the subject of syphilis.

